# On strong KKT type sufficient optimality conditions for multiobjective semi-infinite programming problems with vanishing constraints

**DOI:** 10.1186/s13660-017-1558-x

**Published:** 2017-11-14

**Authors:** Sy-Ming Guu, Yadvendra Singh, Shashi Kant Mishra

**Affiliations:** 1 Graduate Institute of Business and Management, College of Management, Chang Gung University, Kwei-Shan District, Taoyuan City, Taiwan, ROC; 2 Department of Neurology, LinKou Chang Gung Memorial Hospital, Kwei-Shan District, Taoyuan City, Taiwan, ROC; 3 Department of Mathematics, Institute of Science, Banaras Hindu University, Varanasi, 221005 India

**Keywords:** 90C34, 49J52, semi-infinite programming, mathematical programs with vanishing constraints, optimality conditions, generalized convexity

## Abstract

In this paper, we consider a nonsmooth multiobjective semi-infinite programming problem with vanishing constraints (MOSIPVC). We introduce stationary conditions for the MOSIPVCs and establish the strong Karush-Kuhn-Tucker type sufficient optimality conditions for the MOSIPVC under generalized convexity assumptions.

## Introduction

Multiobjective semi-infinite programming problems (MOSIPs) arise when more than one objective function is to be optimized over the feasible region described by an infinite number of constraints. If there is only one objective function in a MOSIP, then it is known as semi-infinite programming problem (SIP). SIPs have played an important role in several areas of modern research, such as transportation theory [1], engineering design [2], robot trajectory planning [3] and control of air pollution [4]. We refer to the books [5, 6] for more details as regards SIPs and their applications and to some recent papers [7–9] for details as regards MOSIPs.

Achtziger and Kanzow [10] introduced the mathematical programs with vanishing constraints (MPVCs) and showed that many problems from structural topology optimization can be reformulated as MPVCs. Hoheisel and Kanzow [11] defined stationary concepts for MPVCs and derived first order sufficient and second order necessary and sufficient optimality conditions for MPVCs. Hoheisel and Kanzow [12] established optimality conditions for weak constraint qualification. Mishra *et al.* [13] obtained various constraint qualifications and established Karush-Kuhn-Tucker (KKT) type necessary optimality conditions for multiobjective MPVCs. We refer to [14–16] and references therein for more details as regards MPVCs.

Recently, the idea of a strong KKT has been used to avoid the case where some of the Lagrange multipliers associated with the components of multiobjective functions vanish. Golestani and Nobakhtian [17] derived the strong KKT optimality conditions for nonsmooth multiobjective optimization. Kanzi [9] established strong KKT optimality conditions for MOSIPs. Pandey and Mishra [18] established the strong KKT type sufficient conditions for nonsmooth MOSIPs with equilibrium constraints.

Motivated by Achtziger and Kanzow [10], Golestani and Nobakhtian [17] and Pandey and Mishra [18], we extend the concept of the strong KKT optimality conditions for the MOSIPs with vanishing constraints (MOSIPVCs) that do not involve any constraint qualification. The paper is organized as follows. In Section 2, we present some known definitions and results which will be used in the sequel. In Section 3, we define stationary points and establish strong KKT type optimality for MOSIPVC. In Section 4, we conclude the results of the paper.

## Definitions and preliminaries

In this paper, we consider the following MOSIPVC:

$$\begin{aligned}& \textstyle\begin{array}{l@{\qquad}l} \mbox{MOSIPVC} & \min f(x):= \bigl( f_{1}(x), f_{2}(x),\ldots, f_{m}(x) \bigr), \\ \mbox{\textit{subject to}}& g_{t}(x)\leq 0,\quad t \in T, \\ & H_{i}(x) \geq 0,\quad i=1,\dots,l, \\ & G_{i}(x)H_{i}(x)\leq 0,\quad i=1,\dots,l, \end{array}\displaystyle \end{aligned}$$

where $f_{i}: \mathbb{R}^{n} \rightarrow \mathbb{R}$, $g_{t}: \mathbb{R}^{n} \rightarrow \mathbb{R}\cup \{+\infty \}$, $G_{i}: \mathbb{R}^{n} \rightarrow \mathbb{R}$, $H_{i}:\mathbb{R}^{n} \rightarrow \mathbb{R}$ are given locally Lipschitz functions and the index set *T* is arbitrary (possibly infinite). Let $M:=\{ x \in \mathbb{R}^{n}: g _{t}(x) \leq 0, t \in T, H_{i}(x) \geq 0, G_{i}(x)H_{i}(x) \leq 0, i=1,\dots,l \}$, denote the feasible set of the MOSIPVC. A point $\bar{x} \in M$ is said to be a weakly efficient solution for the MOSIPVC if there exists no $x \in M$ such that

$$f_{i}(x) < f_{i}(\bar{x}),\quad \forall i=1,2,\dots,m. $$

Let $\bar{x} \in M$. The following index sets will be used in the sequel.

$$\begin{aligned}& T(\bar{x}) := \bigl\{ t \in T: g_{t}(\bar{x})=0\bigr\} , \\& I_{+}(\bar{x}):=\bigl\{ i \in \{1,\dots,l\}: H_{i}( \bar{x})> 0 \bigr\} , \\& I_{0}(\bar{x}) :=\bigl\{ i \in \{1,\dots,l\}: H_{i}( \bar{x})= 0 \bigr\} . \end{aligned}$$

Furthermore, the index set $I_{+}(\bar{x})$ can be divided as follows:

$$\begin{aligned}& I_{+0}(\bar{x}) :=\bigl\{ i \in \{1,\dots,l\}: H_{i}( \bar{x})> 0, G_{i}(x) =0 \bigr\} , \\& I_{+-}(\bar{x}) :=\bigl\{ i \in \{1,\dots,l\}: H_{i}( \bar{x})> 0, G_{i}(x) < 0 \bigr\} . \end{aligned}$$

Similarly, the index set $I_{0}(\bar{x})$ can be partitioned as follows:

$$\begin{aligned}& I_{0+}(\bar{x}) :=\bigl\{ i \in \{1,\dots,l\}: H_{i}( \bar{x})= 0, G_{i}( \bar{x})> 0 \bigr\} , \\& I_{00}(\bar{x}) :=\bigl\{ i \in \{1,\dots,l\}: H_{i}( \bar{x})= 0, G_{i}( \bar{x})= 0\bigr\} , \\& I_{0-}(\bar{x}) :=\bigl\{ i \in \{1,\dots,l\}: H_{i}( \bar{x})= 0, G_{i}( \bar{x})< 0\bigr\} . \end{aligned}$$

The Clarke directional derivative of a locally Lipschitz function $f:\mathbb{R}^{n}\rightarrow \mathbb{R}$ around *x̄* in the direction $v \in \mathbb{R}^{n}$ and the Clarke subdifferential of *f* at *x̄* are, respectively, given by

$$\begin{aligned}& f^{0}(\bar{x};v) := \lim_{x\rightarrow \bar{x}}\sup _{t\downarrow 0} \frac{f(x+tv)-f(x)}{t}, \\& \partial_{c}f(\bar{x}) :=\bigl\{ \xi \in \mathbb{R}^{n}: f^{0}(\bar{x};v) \geq \langle \xi, v\rangle, \forall v \in \mathbb{R}^{n}\bigr\} . \end{aligned}$$

We recall the following results from [19].

### Theorem 2.1

*Let* *f* *and* *g* *be locally Lipschitz from* $\mathbb{R}^{n}$ *to* $\mathbb{R}$ *around* *x̄*. *Then the following properties hold*:

1. $f^{0}(\bar{x};v)= \max \{ \langle \xi, v\rangle: \xi \in \partial _{c} f(\bar{x}), \forall v \in \mathbb{R}^{n}\}$,
2. $\partial_{c}(\lambda f)(\bar{x})=\lambda \partial_{c}f(\bar{x})$, $\forall \lambda \in \mathbb{R}$,
3. $\partial_{c}(f+g)(\bar{x}) \subseteq \partial_{c} f(\bar{x})+ \partial_{c} g(\bar{x})$.

The following definitions and lemma from Kanzi and Nobakhtian [8] will be used in the sequel.

### Definition 2.1

Let $f:\mathbb{R}^{n}\rightarrow \mathbb{R}$ be a locally Lipschitz function around *x̄*. Then

1. *f* is said to be generalized convex at *x̄* if, for each $x \in \mathbb{R}^{n}$ and any $\xi \in \partial_{c} f(\bar{x})$,
  $$f(x) - f(\bar{x}) \geq \langle \xi, x-\bar{x}\rangle, $$
2. *f* is said to be strictly generalized convex at *x̄* if, for each $x \in \mathbb{R}^{n}$, $x\neq \bar{x}$ and any $\xi \in \partial_{c} f( \bar{x})$,
  $$f(x) - f(\bar{x}) > \langle \xi, x-\bar{x}\rangle, $$
3. *f* is said to be generalized quasiconvex at *x̄* if, for each $x \in \mathbb{R}^{n}$ and any $\xi \in \partial_{c} f(\bar{x})$,
  $$f(x) \leq f(\bar{x})\quad \Rightarrow\quad \langle \xi, x-\bar{x}\rangle \leq 0, $$
4. *f* is said to be strictly generalized quasiconvex at *x̄* if, for each $x \in \mathbb{R}^{n}$ and any $\xi \in \partial_{c} f(\bar{x})$,
  $$f(x) \leq f(\bar{x}) \quad \Rightarrow\quad \langle \xi, x-\bar{x}\rangle < 0. $$

### Lemma 2.1

*Let* $f_{0}$ *be strictly generalized convex and* $f_{1},f_{2},\dots,f _{s}$ *be generalized convex function at* *x*. *If* $\lambda_{0} > 0$ *and* $\lambda_{l} \geq 0$ *for* $l=1,\dots,s$, *then* $\sum_{l=1}^{s} \lambda_{l}f_{l}$ *is strictly generalized convex at x*.

## Strong KKT type sufficient optimality conditions

We extend Definitions 2.1 and 2.2 of Hoheisel and Kanzow [11] to the case of the MOSIPVC.

### Definition 3.1

(MOSIPVC S-stationary point)

A feasible point *x̄* of the MOSIPVC is called a MOSIPVC strong (S-)stationary point if there exist Lagrange multipliers $\lambda_{i} > 0$, $i=1,\dots,m$, and $\mu_{t} \geq 0$, $t\in T(\bar{x})$, with $\mu_{t} \neq 0$ for at most finitely many indices and $\eta_{i}^{H}, \eta_{i}^{G} \in \mathbb{R}$, $i=1, \dots, l$ such that the following conditions hold:

$$\begin{aligned}& 0 \in \sum_{i=1}^{m} \lambda_{i} \partial_{c} f_{i}(\bar{x}) + \sum _{t\in T(\bar{x})} \mu_{t} \partial_{c} g_{t} (\bar{x})- \sum_{i=1} ^{l} \eta_{i}^{H} \partial_{c} H_{i}( \bar{x}) + \sum_{i=1}^{l} \eta _{i}^{G} \partial_{c} G_{i}(\bar{x}), \\& \eta_{i}^{H}=0,\quad i \in I_{+}(\bar{x}),\qquad \eta_{i}^{H} \geq 0,\quad i \in I _{0-}(\bar{x})\cup I_{00}(\bar{x}),\qquad \eta_{i}^{H} \in \mathbb{R},\quad i \in I_{0+}(\bar{x}), \\& \eta_{i}^{G}=0,\quad i \in I_{+-}(\bar{x})\cup I_{0}(\bar{x})\cup I_{0+}( \bar{x}),\qquad \eta_{i}^{G} \geq 0, \quad i \in I_{+0}(\bar{x}). \end{aligned}$$

### Definition 3.2

(MOSIPVC M-stationary point)

A feasible point *x̄* of the MOSIPVC is called a MOSIPVC Mordukhovich (M-)stationary point if there exist Lagrange multipliers $\lambda_{i} > 0$, $i=1,\dots,m$, and $\mu_{t} \geq 0$, $t\in T(\bar{x})$, with $\mu_{t} \neq 0$ for at most finitely many indices and $\eta_{i}^{H}$, $\eta_{i}^{G} \in \mathbb{R}$, $i=1, \dots, l$, such that the following conditions hold:

$$\begin{aligned}& 0 \in \sum_{i=1}^{m} \lambda_{i} \partial_{c} f_{i}(\bar{x}) + \sum _{t\in T(\bar{x})} \mu_{t} \partial_{c} g_{t} (\bar{x})- \sum_{i=1} ^{l} \eta_{i}^{H} \partial_{c} H_{i}( \bar{x}) + \sum_{i=1}^{l} \eta _{i}^{G} \partial_{c} G_{i}(\bar{x}), \\& \eta_{i}^{H}=0,\quad i \in I_{+}(\bar{x}),\qquad \eta_{i}^{H} \geq 0, \quad i \in I _{0-}(\bar{x}),\qquad \eta_{i}^{H} \in \mathbb{R}, \quad i \in I_{0+}(\bar{x}), \\& \eta_{i}^{G}=0,\quad i \in I_{+-}(\bar{x})\cup I_{0-}(\bar{x})\cup I_{0+}( \bar{x}),\qquad \eta_{i}^{G} \geq 0, \quad i \in I_{+0}(\bar{x})\cup I_{00}( \bar{x}), \\& \eta^{G}_{i}\cdot \eta^{H}_{i}=0,\quad i \in I_{00}(\bar{x}). \end{aligned}$$

### Remark 3.1

The difference between MOSIPVC M-stationary points and MOSIPVC S-stationary points occurs only for the index set $I_{00}$. For MOSIPVC M-stationary points, $\eta_{i}^{G} \geq 0$ and $\eta_{i}^{H}\cdot\eta_{i} ^{G}=0$ for $i\in I_{00}$, whereas for MOSIPVC S-stationary points, $\eta_{i}^{H} \geq 0$ and $\eta_{i}^{G}= 0$ for $i\in I_{00}$.

In the following theorem, we establish the strong KKT type sufficient optimality result for the MOSIPVC under generalized convexity assumptions.

### Theorem 3.1

*Let* *x̄* *be a MOSIPVC M*-*stationary point*. *Suppose that* $f_{i}$, $i=1,\ldots,m$, $g_{t}$, $t \in T(\bar{x})$, $-H_{i}$, $G_{i}$, $i=1, \dots, l$, *are generalized convex at* *x̄* *on M and at least one of them is strictly generalized convex at* *x̄* *on M*. *Then* *x̄* *is a weakly efficient solution for the MOSIPVC*.

### Proof

Since *x̄* is a MOSIPVC M-stationary point, there exist $\bar{\xi }^{f}_{i} \in \partial_{c}f_{i}(\bar{x})$, $i=1, \dots,m$, $\bar{\xi }^{g}_{t} \in \partial_{c}g_{t}(\bar{x})$, $t \in T(\bar{x})$, and $\bar{\xi }^{H}_{i} \in \partial_{c}H_{i}( \bar{x})$, $\bar{\xi }^{G}_{i} \in \partial_{c}G_{i}(\bar{x})$, $i=1,\ldots,l$, such that

3.1 $$ \sum_{i=1}^{m} \lambda_{i} \bar{\xi }^{f}_{i}+\sum _{t \in T(\bar{x})} \mu_{t} \bar{\xi }^{g}_{t}- \sum_{i=1} ^{l}\eta^{H}_{i} \bar{\xi }^{H}_{i}+\sum_{i=1}^{l} \eta^{G} _{i} \bar{\xi }^{G}_{i}=0. $$

Suppose on the contrary that *x̄* is not a weakly efficient solution for the MOSIPVC, that is, there exists $\tilde{x} \in M$, such that

$$f_{i}(\tilde{x}) < f_{i}(\bar{x})\quad \mbox{for all } i=1,\dots,m. $$

From the MOSIPVC M-stationary point, we have $\lambda_{i}> 0$ for $i=1,\dots, m$. Thus, we get

3.2 $$ \sum_{i=1}^{m} \lambda_{i} f_{i}(\tilde{x}) < \sum _{i=1} ^{m} \lambda_{i} f_{i}( \bar{x}). $$

Since *x̄* is a MOSIPVC M-stationary point and *x̃* is a feasible point of the MOSIPVC, we have

$$\begin{aligned}& g_{t}(\tilde{x}) < 0,\qquad \mu_{t} \geq 0, \quad t \in T( \bar{x}), \\& -H_{i}(\tilde{x}) < 0,\qquad \eta^{H}_{i} \geq 0,\quad i \in I_{0-}(\bar{x}) \cup I_{+}(\bar{x}), \\& -H_{i}(\tilde{x}) = 0,\qquad \eta^{H} \in \mathbb{R},\quad i \in I_{0+}( \bar{x}), \\& G_{i}(\tilde{x}) > 0,\qquad \eta^{G}=0,\quad i \in I_{+-}(\bar{x}) \cup I _{0-}(\bar{x}) \cup I_{0+}( \bar{x}), \\& G_{i}(\tilde{x}) \leq 0,\qquad \eta^{G} > 0,\quad i \in I_{00}(\bar{x}) \cup I_{+0}(\bar{x}), \end{aligned}$$

which implies that

3.3 $$\begin{aligned}& \sum_{t\in T(\bar{x})} \mu_{t}g_{t}( \tilde{x})-\sum_{i=1} ^{l} \eta^{H}_{i} H_{i}(\tilde{x})+ \sum _{i=1}^{l} \eta^{G} _{i} G_{i}(\tilde{x}) \\& \quad \leq \sum_{t\in T(\bar{x})} \mu_{t}g_{t}( \bar{x}) -\sum_{i=1}^{l} \eta^{H}_{i} H_{i}(\bar{x})+ \sum_{i=1} ^{l} \eta^{G}_{i} G_{i}(\bar{x}). \end{aligned}$$

From (3.2) and (3.3), we have

3.4 $$\begin{aligned}& \sum_{i=1}^{m} \lambda_{i} f_{i}(\tilde{x})+ \sum _{t\in T(\bar{x})} \mu_{t}g_{t}(\tilde{x})-\sum _{i=1} ^{l} \eta^{H}_{i} H_{i}(\tilde{x})+ \sum_{i=1}^{l} \eta^{G} _{i} G_{i}(\tilde{x}) \\& \quad < \sum_{i=1}^{m} \lambda_{i} f_{i}(\bar{x})+ \sum_{t\in T(\bar{x})} \mu_{t}g_{t}(\bar{x})-\sum_{i=1}^{l} \eta^{H}_{i} H_{i}(\bar{x})+ \sum _{i=1}^{l} \eta^{G}_{i} G_{i}( \bar{x}). \end{aligned}$$

It follows from Lemma 2.1 that $\sum_{i=1}^{m} \lambda _{i} f_{i}({x})+ \sum_{t\in T(\bar{x})} \mu_{t}g_{t}({x})- \sum_{i=1}^{l} \eta^{H}_{i} H_{i}({x})+ \sum_{i=1}^{l} \eta^{G}_{i} G_{i}({x})$ is a strictly generalized convex function at *x̄* on *M*. Hence,

3.5 $$\begin{aligned} 0 =&\sum_{i=1}^{m} \lambda_{i} \bar{\xi }^{f}_{i}+\sum _{t \in T(\bar{x})} \mu_{t} \bar{\xi }^{g}_{t}- \sum_{i=1} ^{l}\eta^{H}_{i} \bar{\xi }^{H}_{i} \\ &{}+\sum_{i=1}^{l} \eta^{G} _{i} \bar{\xi }^{G}_{i} \in \partial_{c} \Biggl( \sum_{i=1}^{m} \lambda_{i} f_{i}({\bar{x}})+ \sum_{t\in T(\bar{x})} \mu_{t}g_{t}( \bar{x})-\sum_{i=1}^{l} \eta^{H}_{i} H_{i}(\bar{x})+ \sum_{i=1}^{l} \eta^{G}_{i} G_{i}(\bar{x}) \Biggr). \end{aligned}$$

Therefore, from (3.1), (3.4) and (3.5), we obtain

$$0 > \Biggl\langle \sum_{i=1}^{m} \lambda_{i} \bar{\xi }^{f}_{i}+ \sum _{t \in T(\bar{x})} \mu_{t} \bar{\xi }^{g}_{t}- \sum_{i=1}^{l}\eta^{H}_{i} \bar{\xi }^{H}_{i}+\sum_{i=1} ^{l} \eta^{G}_{i} \bar{\xi }^{G}_{i}, \tilde{x}-\bar{x} \Biggr\rangle = \langle 0, \tilde{x}-\bar{x} \rangle. $$

Thus, we arrive at a contradiction and hence the result. □

The following result is a direct consequence of Theorem 3.1, where the MOSIPVC M-stationary point is replaced by a MOSIPVC S-stationary point.

### Corollary 3.1

*Let* *x̄* *be a MOSIPVC S*-*stationary point*. *Suppose that* $f_{i}$, $i=1,\ldots,m$, $g_{t}$, $t \in T(\bar{x})$, $-H_{i}$, $G_{i}$, $i=1, \dots, l$, *are generalized convex at* *x̄* *on M and at least one of them is strictly generalized convex at* *x̄* *on M*. *Then* *x̄* *is a weakly efficient solution for the MOSIPVC*.

The strong KKT type sufficient condition for the MOSIPVC given in Theorem 3.1 can be obtained under further relaxations on generalized convexity requirements.

### Theorem 3.2

*Let* *x̄* *be a MOSIPVC M*-*stationary point*. *Suppose that* $f_{i}$, $i=1,\ldots,m$, $g_{t}$, $t \in T(\bar{x})$, $-H_{i}$, $G_{i}$, $i=1, \dots, l$, *are generalized quasiconvex at* *x̄* *on M and at least one of them is strictly generalized quasiconvex at* *x̄* *on M*. *Then* *x̄* *is a weakly efficient solution for the MOSIPVC*.

The following example satisfies the assumptions of Theorem 3.1.

### Example 3.1

Consider the following problem in $\mathbb{R}^{2}$:

3.6 $$\begin{aligned}& \textstyle\begin{array}{l@{\qquad}l} \min &f(x)=\bigl(x_{1}^{2}, \vert x_{1}\vert +\vert x_{2}\vert \bigr), \\ \text{s. t.} & g_{t}(x)=-t x_{1} \leq 0,\quad t\in \mathbb{N}, \\ & H(x)=x_{1} \geq 0, \\ & H(x)G(x)= x_{1}\bigl(\vert x_{1}\vert +x_{2}\bigr) \leq 0. \end{array}\displaystyle \end{aligned}$$

**Figure 1:**
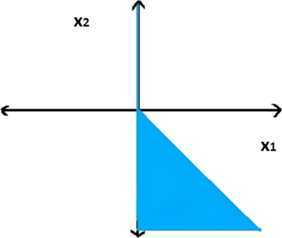
**Plot of the feasible region of MOSIPVC (** **3.6** **).**

Note that $f_{1}(x)=\vert x_{1}\vert $, $f_{2}(x)=\vert x_{1}\vert +\vert x_{2}\vert $ and the feasible region of the MOSIPVC (3.6) is given by

$$M=\bigl\{ (x_{1},x_{2}) \in \mathbb{R}^{2} : -t x_{1} \leq 0, t\in \mathbb{N},x_{1} \geq 0, x_{1}\bigl(\vert x_{1}\vert +x_{2}\bigr) \leq 0 \bigr\} , $$

which is represented by the shaded region in Figure 1.

It is easy to see that $\bar{x}= ( 0,0 ) $ is a feasible point of the problem, $T(\bar{x})=\mathbb{N}$ and $I_{00}(\bar{x})=\{1\}$. The feasible point *x̄* is a MOSIPVC M-stationary point with $\lambda_{1}>0$, $\lambda_{2}=1$, $\mu_{1}=1$, $\mu_{2}=\frac{1}{2}$, $\mu_{3}= \mu_{4}=\cdots=0$, $\eta^{H}=-1$, $\eta^{G}=0$, $\xi^{f_{1}}=(0,0) \in \partial_{c} f_{1}(\bar{x})=\{(0,0)\}$, $\xi^{f_{2}}=(1,0)\in \partial_{c} f_{2}(\bar{x})=[-1,1]\times [-1,1]$, $\xi_{1}^{g_{t}}=(-t,0)\in \partial _{c} g_{t}(\bar{x})=\{(-t,0)\}$, $\xi^{H}=(1,0) \in \partial_{c} H( \bar{x})=\{(1,0)\}$ and $\xi^{G}=(0,1) \in \partial_{c} G(\bar{x})=[-1,1] \times \{1\}$.

The strong KKT type sufficient optimality condition for the MOSIPVC can also be obtained in the following way.

### Theorem 3.3

*Let* *x̄* *be a MOSIPVC M*-*stationary point*. *Suppose that each* $f_{i}$, $i=1,\ldots,m$, *is generalized convex at* *x̄* *on M and* $\sum_{t\in T(\bar{x})} \mu_{t}g_{t}({x})-\sum_{i=1} ^{l} \eta^{H}_{i} H_{i}({x})+ \sum_{i=1}^{l} \eta^{G}_{i} G _{i}({x})$ *is generalized convex at* *x̄* *on M*. *Then* *x̄* *is a weakly efficient solution for the MOSIPVC*.

**Proof** Suppose on the contrary that *x̄* is not a weakly efficient solution for the MOSIPVC, that is, there exists a feasible point *x̃* such that

$$f_{i}(\tilde{x}) < f_{i}(\bar{x}),\quad \forall i=1,\ldots,m. $$

By strictly generalized convexity of $f_{i}$, we have

3.7 $$ \bigl\langle \xi_{i}^{f}, \tilde{x}-\bar{x}\bigr\rangle < 0, \quad \forall \xi_{i} ^{f} \in \partial_{c}f_{i}( \bar{x}), i=1,\ldots,m. $$

From the M-stationary condition, we have $\lambda_{i} > 0$, $i=1,\ldots,m$. Thus, we get

3.8 $$ \Biggl\langle \sum_{i=1}^{m} \lambda_{i} \xi_{i}^{f}, \tilde{x}- \bar{x} \Biggr\rangle < 0. $$

Since *x̄* is a MOSIPVC M-stationary point, from (3.1) and (3.8), we have

3.9 $$ \Biggl\langle \sum_{t \in T(\bar{x})} \mu_{t} \bar{\xi }^{g}_{t}- \sum _{i=1}^{l}\eta^{H}_{i} \bar{\xi }^{H}_{i}+\sum_{i=1} ^{l} \eta^{G}_{i} \bar{\xi }^{G}_{i}, \tilde{x}-\bar{x} \Biggr\rangle > 0. $$

From (3.3), we have

3.10 $$\begin{aligned}& \sum_{t\in T(\bar{x})} \mu_{t}g_{t}( \tilde{x})-\sum_{i=1} ^{l} \eta^{H}_{i} H_{i}(\tilde{x})+ \sum _{i=1}^{l} \eta^{G} _{i} G_{i}(\tilde{x}) \\& \quad \leq \sum_{t\in T(\bar{x})} \mu_{t}g_{t}( \bar{x}) -\sum_{i=1}^{l} \eta^{H}_{i} H_{i}(\bar{x})+ \sum_{i=1} ^{l} \eta^{G}_{i} G_{i}(\bar{x}). \end{aligned}$$

From the generalized convexity of $\sum_{t\in T(\bar{x})} \mu _{t}g_{t}({x})-\sum_{i=1}^{l} \eta^{H}_{i} H_{i}({x})+ \sum_{i=1}^{l} \eta^{G}_{i} G_{i}({x})$, at *x̄* on *M*, we get

3.11 $$ \Biggl\langle \sum_{t \in T(\bar{x})} \mu_{t} \bar{\xi }^{g}_{t}- \sum_{i=1}^{l} \eta^{H}_{i} \bar{\xi }^{H}_{i}+\sum _{i=1} ^{l} \eta^{G}_{i} \bar{\xi }^{G}_{i}, \tilde{x}-\bar{x} \Biggr\rangle \leq 0, $$

which contradicts (3.9). Hence, *x̄* is a weakly efficient solution of the MOSIPVC and the proof is complete.

The following result is a direct consequence of Theorem 3.3, where the MOSIPVC M-stationary point is replaced by a MOSIPVC S-stationary point.

### Corollary 3.2

*Let* *x̄* *be a MOSIPVC S*-*stationary point*. *Suppose that each* $f_{i}$, $i=1,\ldots,m$ *is generalized convex and* $\sum_{t\in T(\bar{x})} \mu_{t}g_{t}({x})-\sum_{i=1}^{l} \eta ^{H}_{i} H_{i}({x})+ \sum_{i=1}^{l} \eta^{G}_{i} G_{i}({x})$ *is generalized convex at* *x̄* *on M*. *Then* *x̄* *is a weakly efficient solution for the MOSIPVC*.

The following example satisfies the assumptions of Theorem 3.3.

### Example 3.2

Consider the following problem in $\mathbb{R}^{2}$:

3.12 $$\begin{aligned}& \textstyle\begin{array}{l@{\qquad}l} \min &f(x)=\bigl(\vert x_{1}\vert , \vert x_{2}\vert \bigr), \\ \mbox{s.t.} & g_{t}(x)=-t x_{1}^{3} \leq 0, \quad t \in \mathbb{N}, \\ & H(x)=x_{1}^{3}+x_{2} \geq 0, \\ & G(x)H(x)= \vert x_{1}\vert \bigl(x_{1}^{3}+x_{2} \bigr) \leq 0. \end{array}\displaystyle \end{aligned}$$

**Figure 2:**
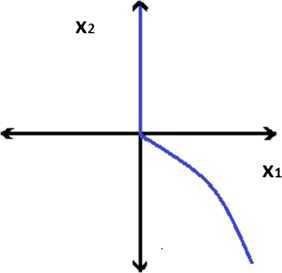
**Plot of the feasible region of MOSIPVC (** **3.12** **).**

Note that $f_{1}(x)=\vert x_{1}\vert $, $f_{2}(x)=\vert x_{2}\vert $ and the feasible region of the MOSIPVC (3.12) is given by

$$M=\bigl\{ (x_{1},x_{2}) \in \mathbb{R}^{2} : -t x_{1}^{3} \leq 0, t \in \mathbb{N}, x_{1}^{3}+x_{2} \geq 0, \vert x_{1}\vert \bigl(x_{1}^{3}+x_{2} \bigr) \leq 0 \bigr\} , $$

which is represented by the shaded region in Figure 2.

It is easy to see that $\bar{x}= ( 0,0 ) $ is a feasible point of the problem, $T(\bar{x})=\mathbb{N}$ and $I_{00}(\bar{x})=\{1\}$. The feasible point *x̄* is a MOSIPVC M-stationary point with $\lambda_{1} > 0$, $\lambda_{2}=1$, $\mu_{1}=1$, $\mu_{2}=\mu_{3}=\cdots=0$, $\eta ^{H}_{1}=-1$, $\eta^{G}_{1}=0$, $\xi^{f_{1}}=(0,0) \in \partial_{c} f _{1}(\bar{x})=[-1,1]\times \{0\}$, $\xi^{f_{2}}=(0,-1)\in \partial_{c} f _{2}(\bar{x})=\{0\}\times [-1,1]$, $\xi_{1}^{g_{t}}=(0,0)\in \partial_{c} g_{t}(\bar{x})=\{(0,0)\}$, $\xi^{H}=(0,1) \in \partial_{c} H(\bar{x})= \{(0,1)\}$ and $\xi^{G}=(1,0) \in \partial_{c} G(\bar{x})=[-1,1] \times \{0\}$. Also, $\mu_{1}g_{1}({x})+\mu_{2}g_{2}({x})+\cdots- \eta ^{H}_{1} H({x})+ \eta^{G}_{1} G({x})=-x_{1}^{3}+x_{1}^{3}+x_{2}-0$. $\vert x _{1}\vert = x_{2}$ is generalized convex at *x̄* on *M*.

## Results and discussion

In this paper, we consider a MOSIPVC. We introduce stationary conditions for the MOSIPVC and establish the strong KKT type sufficient optimality conditions for the MOSIPVC under generalized convexity assumptions. We extend the concept of the strong KKT optimality conditions for the MOSIPVC that do not involve any constraint qualification. Furthermore, the results of this paper may be extended to strong KKT type necessary optimality conditions for the MOSIPVC involving constraint qualification.
